# Long term corrosion estimation of carbon steel, titanium and its alloy in backfill material of compacted bentonite for nuclear waste repository

**DOI:** 10.1038/s41598-019-39751-9

**Published:** 2019-03-01

**Authors:** Qichao Zhang, Min Zheng, Yanliang Huang, Hans Joerg Kunte, Xiutong Wang, Yuemiao Liu, Chuanbo Zheng

**Affiliations:** 1 Institute of Oceanology, Chinese Academy of Sciences, Qingdao, 266071 China; 2 University of Chinese Academy of Sciences, Beijing, 100049 China; 3 Bundesanstalt für Materialforschung und –prüfung, Berlin, 12205 Germany; 4 Beijing Research Institute of Uranium Geology, Beijing, 100029 China; 5 School of Materials Science and Engineering, Jiangsu University of Science and Technology, Zhenjiang, 212003 China; 6 Open Studio for Marine Corrosion and Protection, Qingdao National Laboratory for Marine Science and Technology, Qingdao, 266237 China

## Abstract

The container of high-level radioactive waste (HLRW) being in deep geological disposal, the backfill material is needed to serve as the second defense for HLRW and the highly compacted bentonite is generally selected. As the time goes, the underground water will infiltrate the backfill, causing the corrosion of materials for the building of containers in the formed electrolyte. Carbon steel, titanium and its alloy are the potential candidate materials for the fabrication of HLRW containers. The current investigation aims at assessing the safety of HLRW container in deep geological disposal for hundreds of thousands of years and facilitating the material selection for future container fabrication by estimating their corrosion behavior in compacted bentonite with a series of moisture content at different temperatures through electrochemical methods including open circuit potential (OCP), electrochemical impedance spectroscopy (EIS) and potentiodynamic polarization curve (PC) measurements. The corrosion rates were estimated for a carbon steel, a pure titanium and a titanium alloy in compacted Gaomiaozi Bentonite infiltrated with simulated underground water in Beishan area of China over an expected disposal period up to 10^6^ years respectively, showing that titanium and its alloy are more reliable materials for building HLRW containers than carbon steel.

## Introduction

Nuclear techniques are widely used by many countries in a lot of fields including energy, medical science, manufacture, agriculture and so on^1^. However, while we enjoy the convenience nuclear techniques bring us, they produce large amounts of HLRW as well, containing various radioactive elements with characteristics of strong radioactivity of calorific value, high toxicity and long half-time that are harmful to human body and biosphere. It is said that over 705 thousand tons of nuclear waste await processing globally, while the total burden of HLRW produced by nuclear power stations in China was about 1000 tons until the year 2010^2,3^. The public only has concerns about nuclear power plants, mostly because of fear of a nuclear leak. For example, the Fukushima Daiichi nuclear disaster has been the second most serious crisis of a nuclear power plant in the human history^4^. However, compared to one percent (or perhaps lower) probability of nuclear leakage, the 100% existence of deadly nuclear waste with radioactive pollution of up to one hundred thousand years or even millions of years is the greatest harm and disaster of future generations. Therefore, solving the problem of HLRW disposal efficiently and reasonably is the most difficult challenge for each country that wants to develop the nuclear power rapidly^5^. Methods for disposing nuclear wastes have been under study for more than 30 years^6^. Deep geological disposal with a ‘multi-barrier system’ consisting container, backfill materials and surrounding rocks is currently generally accepted by many countries^7–10^. Once the container is damaged due to corrosion, surface waters and underground waters play a role in the transportation of radionuclides in water bodies^11^, causing harm to humans. So the waste container serving as the first barrier to prevent HLRW from migrating into biosphere is of great importance. Corrosion effect of HLRW container is one of the most important problems needing to be solved in the HLRW disposal. Apart from corrosion effect, many problems influencing HLRW disposal are to be solved. For example, radiation damage of radioactive waste forms can result in changes in volume, leach rate, stored energy, structure/microstructure and mechanical properties^12^. Radiation not only affects the immobilization of HLRW, but also affects the environment around the container mainly producing chemical changes in the groundwater. From a geological point of view, the geological barrier plays an important role in retarding access of groundwater to the waste form and delaying migration of contaminated groundwater from the waste package to the biosphere^13^. Furthermore, there are microbial effects on repository integrity, such as direct biodeteriation of repository material, direct radionuclide uptake by microorganism making migration enhance, disruption of surfaces on metals^14^.

Materials like low carbon steel, titanium, titanium alloy, copper and nickel base alloy are competitive candidates for building containers at present^15–19^. Japan, Canada, France, Belgium and Switzerland all take carbon steel as container material^20^. Q235 steel is the common economical and practical material for engineering construction in China. Titanium and its alloy are important structural materials due to their combination of low density, high strength and ability to withstand extremes of temperature^21^, making them generally regarded as effective corrosion resistant materials. As for backfill materials, there are two options chosen by different countries. For example, Sweden, Finland and Canada^22–24^ take bentonite as backfill materials while Belgium^25,26^ uses concrete. Meanwhile, as time goes by, the underground water will infiltrate the backfill materials toward the interface of the container continuously and finally the backfill will be saturated, forming an environment where the corrosion of container could happen. Therefore, it is necessary to study the corrosion behavior of the container in bentonite under deep geological disposal conditions and to estimate the corrosion rates in geological time scale.

**Figure 1:**
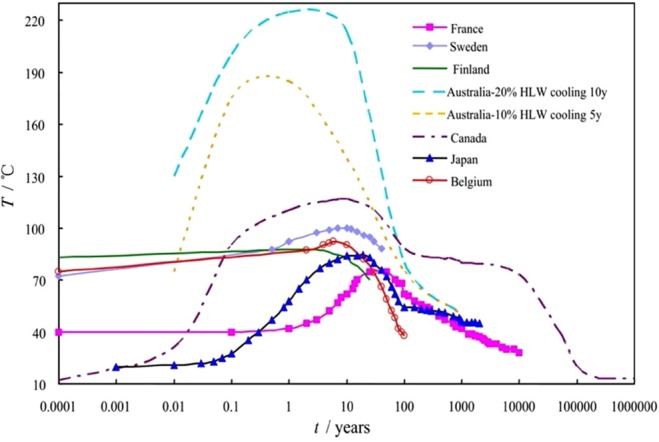
Temperature simulation for near the HLRW containers from various countries.

**Figure 2:**
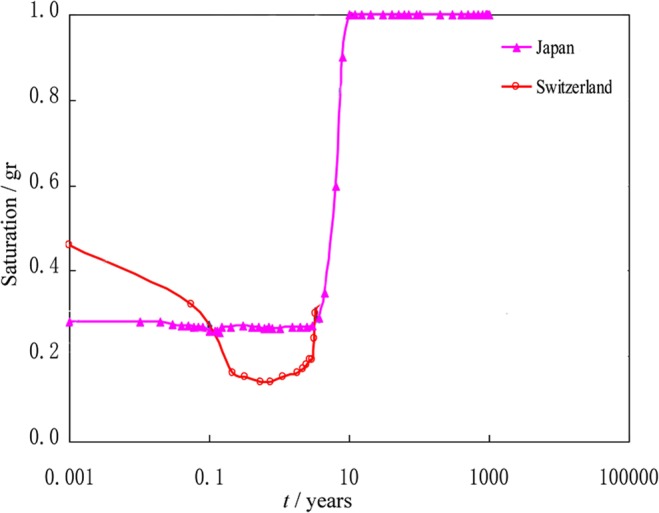
Bentonite saturability simulation in the near-field of waste container in short and long terms.

**Figure 3:**
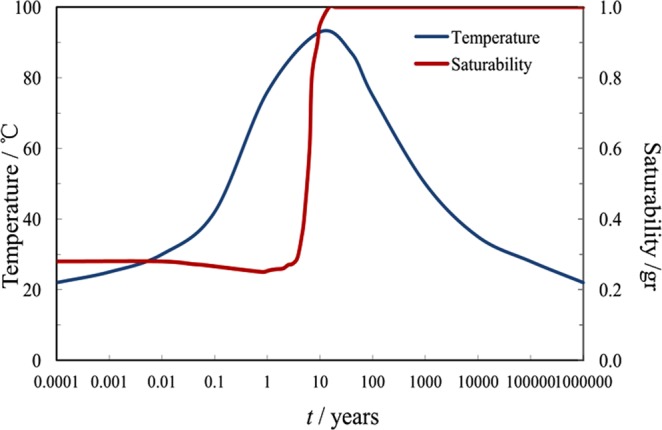
The simulated evolution of temperature and bentonite saturability near the HLRW containers for China.

Because the HLRW during decay is a heat source which can last decades or even hundreds of thousands of years, the long-term variation rule of temperature cannot be observed directly through experiments. The evolution law of the container’s surface temperature and degree of water saturation are calculated by model simulation. The temperature and bentonite saturability simulation models near to the HLRW container published in various countries were summarized in Figs 1 and 2, respectively^22,23,25–30^, from these data the model of China was desired and is shown in Fig. 3. The saturation of water will be reached in about 10 years after disposal, and the water content will be almost constant in the following years. In the meantime, the temperature will also reach the maximum just after bentonite’s reaching its saturation, and then it gradually decreases

Because of good adsorbability and very low permeability^31^, bentonite was considered as backfill material in China with Gaomiaozi bentonite as the most accepted candidate^32^. Moreover, the Beishan area was selected as the most prospective site for a disposal repository of HLRW in China, the compacted Gaomiaozhi bentonite with different percentage of simulated groundwater of Beishan area was used as corrosive media in this paper^33^. The work of this paper focuses on assessment of safety about HLRW container by the estimation of the corrosion behavior of carbon steel, titanium and its alloy in compacted bentonite infiltrated with simulated underground water of Beishan area of China in a geological time scale to facilitate the optimal selection of container materials and design. Meanwhile, our work can be used as a reference for modeling the corrosion life prediction of materials in chloride-containing environment. For many bridges, the concrete decks will need to be rehabilitated before other components of the bridge. Chloride-induced corrosion of the reinforcing steel is known to be a major cause of premature rehabilitation of bridge decks^34^. Similarly, according to the chloride initiation concentration and time for corrosion damage with time evolution, model for the chloride-induced corrosion service life of bridge decks can be acquired. For underground pipelines, combined with a mechanical probabilistic model, corrosion remaining life of underground pipelines can also be predicted^35^. Titanium has been used for piping in desalination plants and for heat exchangers and condensers in marine environments which is close to the condition of our research^36^. Apart from applications in engineering, titanium metal and its alloys are used in dental and orthopedic implants on account of their excellent corrosion resistance, biocompatibility and osseointegration behavior^37^. Because human body contains chloride ions, the electrochemical method in our research can also be used to study the corrosion behavior of titanium and its alloy implanted in human body and to predict the corrosion life.

## Results and Discussion

### Open circuit potential

**Figure 4:**
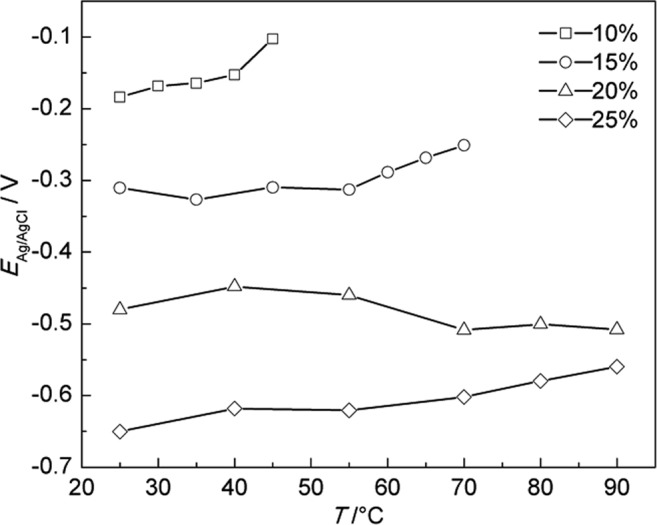
Average OCP variation of A283-D steel in highly compacted bentonite with different water content during temperature increase stage.

Figure 4 shows the average OCP variation of A283-D steel in highly compacted bentonite with different water content during temperature increase stage. It is obvious that OCP of A283-D steel shift to the positive with the decrease of water content, which is directly resulted from less water content’s causing of the less mobility of ions that affect the corrosion process. In other words, the conductivity of bentonite decreases with the decrease of water content, which was proved by Rhoades and Seladji *et al*.^38,39^.

**Figure 5:**
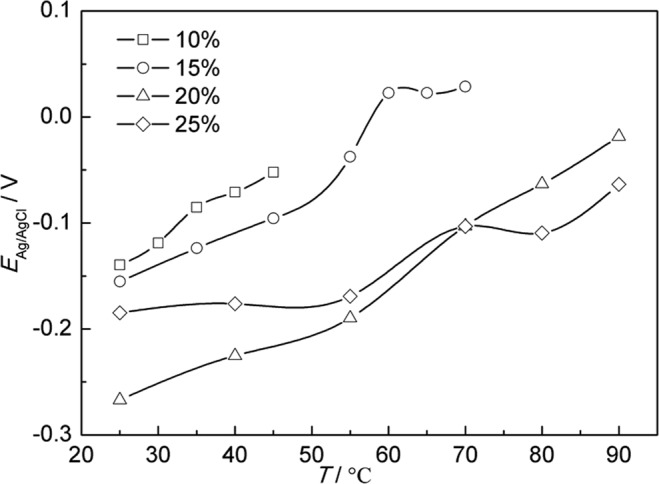
Average OCP variation of Ti grade 2 in highly compacted bentonite with different water content during temperature increase stage.

Figure 5 is average OCP variation of Ti grade 2 in highly compacted bentonite with different water content during temperature increase stage, which is also similar in trend with A283-D steel. However, the OCP of Ti grade 2 is always more positive than A283-D, indicating that the corrosion rate tendency of Ti grade 2 is smaller than that of A283-D under the same conditions.

**Figure 6:**
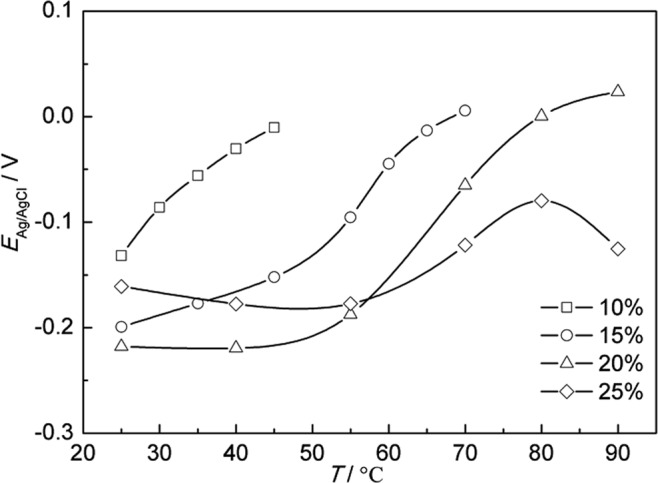
Average OCP variation of Ti grade 16 in highly compacted bentonite with different water content during temperature increase stage.

Figure 6 shows the average OCP variation of Ti grade 16 in highly compacted bentonites with different water content during temperature increase stage. The OCP trend for the water content of 25% is inconsistent with others below 60 °C. Two factors may attribute to the difference. One is that there is a higher coverage of continuous electrolyte film formed on the surface of Ti grade 16 in bentonite with 25% water content, making the corrosive environment at the bentonite/Ti grade 16 interface close to the condition of complete water. Another factor is the existence of Pd in Ti grade 16. The addition of noble metal Pd causes the reduction in the oxygen reduction overpotential and the positive shift of the corrosion potential^18^.

### EIS measurements

**Figure 7:**
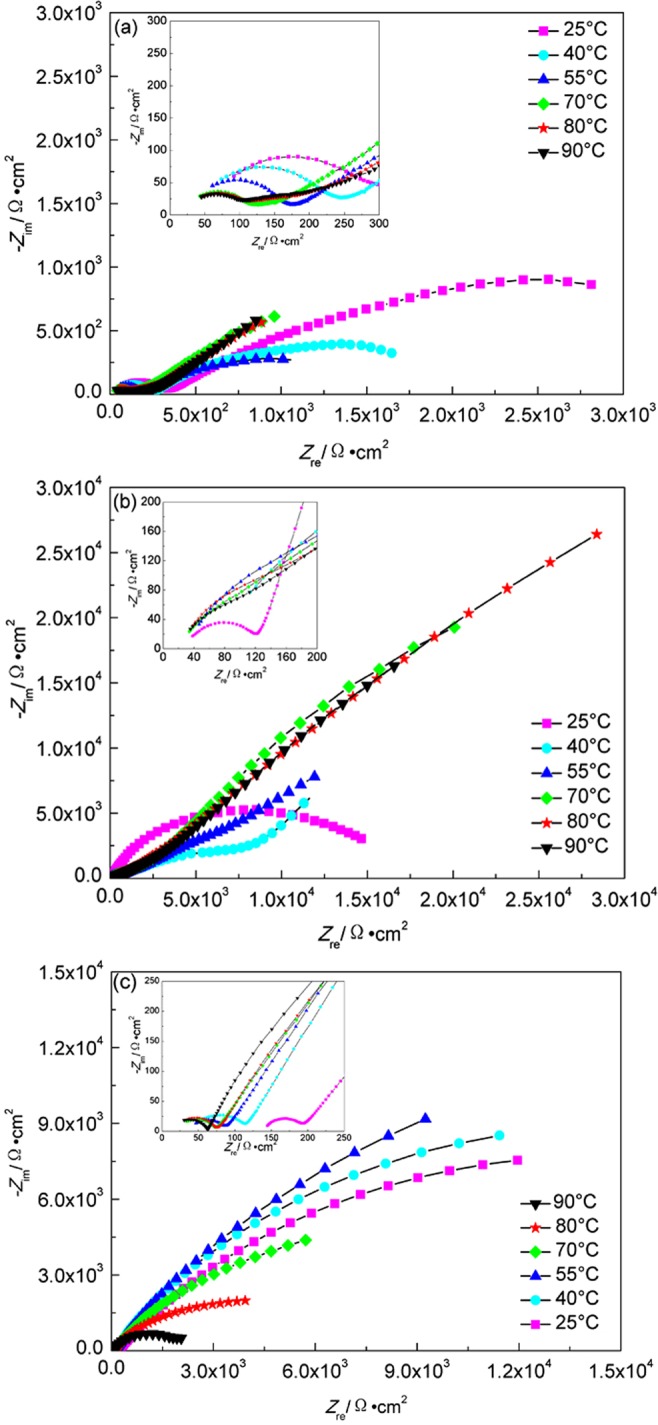
EIS plots of A283-D steel in highly compacted bentonite at different temperatures. (**a**) 20% water, temperature increase; (**b**) 25% water, temperature increase; (**c**) 30% water, temperature decrease.

Figure 7(a,b) shows EIS plots of A283-D steel in compacted bentonite with water content of 20% and 25% respectively. The gradient temperature increase was adopted to simulate the initial stage of temperature variation after disposal. The characteristics of obvious diffusion control appear at 55 °C and 40 °C for water content of 20% and 25% respectively. Lower water content bentonite restricts the diffusion processes of both cathodic and anodic reactions. Here, the basic electrochemical reactions for the corrosion of A283-D steel are as follows^40^:

1 $${\rm{F}}{\rm{e}}-2{{\rm{e}}}^{-}\to {{\rm{F}}{\rm{e}}}^{2+}$$

2 $$2{{\rm{H}}}^{+}+2{{\rm{e}}}^{-}\to {{\rm{H}}}_{2}$$

3 $$2{{\rm{H}}}_{2}{\rm{O}}+2{{\rm{e}}}^{-}\to 2{{\rm{O}}{\rm{H}}}^{-}+{{\rm{H}}}_{2}$$

4 $$1/2{{\rm{O}}}_{2}+{{\rm{H}}}_{2}{\rm{O}}+2{{\rm{e}}}^{-}\to 2{{\rm{O}}{\rm{H}}}^{-}$$

Reaction (1), the anodic reaction, must be balanced by one or more of the cathodic reactions. At the beginning of experiment, the main cathodic reaction was reaction (4) because bentonite contains a certain amount of oxygen. Once the oxygen was exhausted, reaction (3) became the dominant reaction. Because of alkaline environment, Fe^2+^ normally reacts with OH^−^ to form Fe(OH)_2_. However, if the temperature is above 60 °C, the Schikorr reaction^41^ happens:

5 $$3{\rm{F}}{\rm{e}}{({\rm{O}}{\rm{H}})}_{2}\to {{\rm{F}}{\rm{e}}}_{3}{{\rm{O}}}_{4}+2{{\rm{H}}}_{2}{\rm{O}}+{{\rm{H}}}_{2}$$

Therefore, the corrosion product contains Fe(OH)_2_ and Fe_3_O_4_. A larger concentration gradient is formed on the surface of the sample, and the diffusion step becomes a factor controlling the corrosion process

**Figure 8:**
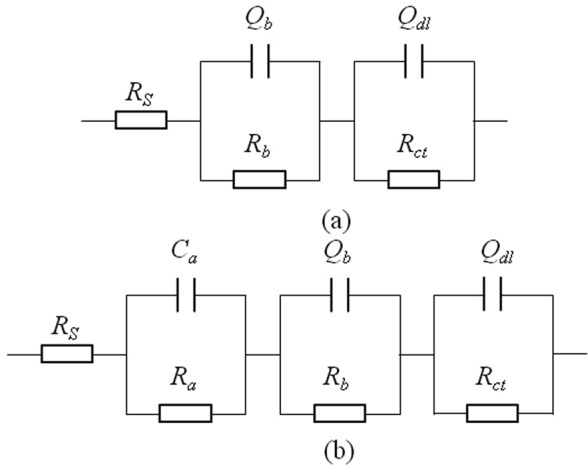
Equivalent circuits of specimens in highly compacted bentonite saturated with water. (**a**) For A283-D steel and Ti grade 2; (**b**) For Ti grade 16. *R*_*s*_, the corrosive medium resistance; *Q*_*b*_, the condensance of corrosion product layer; *R*_*b*_, the resistance of corrosion product layer; *Q*_*dl*_, the electrical double-layer capacitor between the surface of specimen and solution; *R*_*ct*_, the charge transfer resistance; *C*_*a*_, capacitance of recrystallized film; *R*_*a*_, resistance of recrystallized film.

Figure 7(c) shows EIS plots of A283-D steel at different temperatures in highly compacted bentonite with saturated water (30%). The gradient temperature decrease was adopted to simulate the cooling process after the climax of water saturation in bentonite. The radius of condensance arc at low frequency increases with the temperature decrease. The oxygen content is fairly low in highly compacted bentonite saturated with water, therefore, it is not the main factor affecting the corrosion processes at different temperatures. Due to the gradual corrosion products formation process, the initially generated corrosion products are of only small amount and the corrosion products layer is fairly thin, no obvious protective effect on the metal substrate exists. While higher temperature accelerates the corrosion reactions, thus the initial smaller radius of capacitive arc at higher temperature was observed. As the temperature decreases, the combined reduction effects of temperature on electrochemistry reaction rate and the thickening of the accumulated corrosion products layer show increasing in radius of capacitive arc. But below 55 °C, it seems that the radius of the capacitive arc starts to decrease gradually. The concentration of Cl^−^ and SO_4_^2−^ at the interface between container and the bentonite is consistently at a higher level because of their high concentration in underground water, and they can also be regenerated and accumulated on the surface of container^39^. Of these two species, Cl^−^ can weaken the passive film formed on the surface of metal and penetrate through the corrosion product layer, forming soluble corrosion product. Although further decrease of temperature below 55 °C reduces the electrochemical reaction rate, if the weakening effect of Cl^−^ on the corrosion products layer is stronger than that of temperature on the reduction of electrochemical reaction, the corrosion process as a whole will be enhanced, resulting in a smaller capacitive arc radius. It can also be found that the change of capacitive arc radius with temperature at higher temperature above 55 °C is larger than that below, indicating that the corrosion rate of container tends to be steady in the later period of deep geological disposal. The equivalent circuit shown in Fig. 8(a) was used to fit the EIS data of A283-D specimen, where *R*_*s*_ is the corrosion medium resistance, *Q*_*b*_ is the condensance of corrosion product layer, *R*_*b*_ is the resistance of corrosion product layer, *Q*_*dl*_ is the electrical double-layer capacitor between the surface of specimen and solution, *R*_*ct*_ is the charge transfer resistance. The parameters fitted by ZSimpWin software are shown in Table 1. It can be seen from Table 1 that the film resistance increases gradually with the continuous accumulation of corrosion products formed on the surface of container. The change of charge transfer resistance with temperature roughly conforms with that of capacitive arc radius.

**Table 1:** Fitted EIS results of A283-D steel in highly compacted bentonite with 30% water content at different temperatures.

Temperature(°C)	*R*_*s*_(Ω·cm^2^)	*R*_*b*_(Ω·cm^2^)	*R*_*ct*_(Ω·cm^2^)
90	46.52	15.18	2.14 × 10^3^
80	57.51	16.61	5.88 × 10^3^
70	51.71	21.02	1.47 × 10^4^
55	48.40	37.40	3.80 × 10^4^
40	71.28	40.53	2.82 × 10^4^
25	139.90	51.02	2.71 × 10^4^

**Figure 9:**
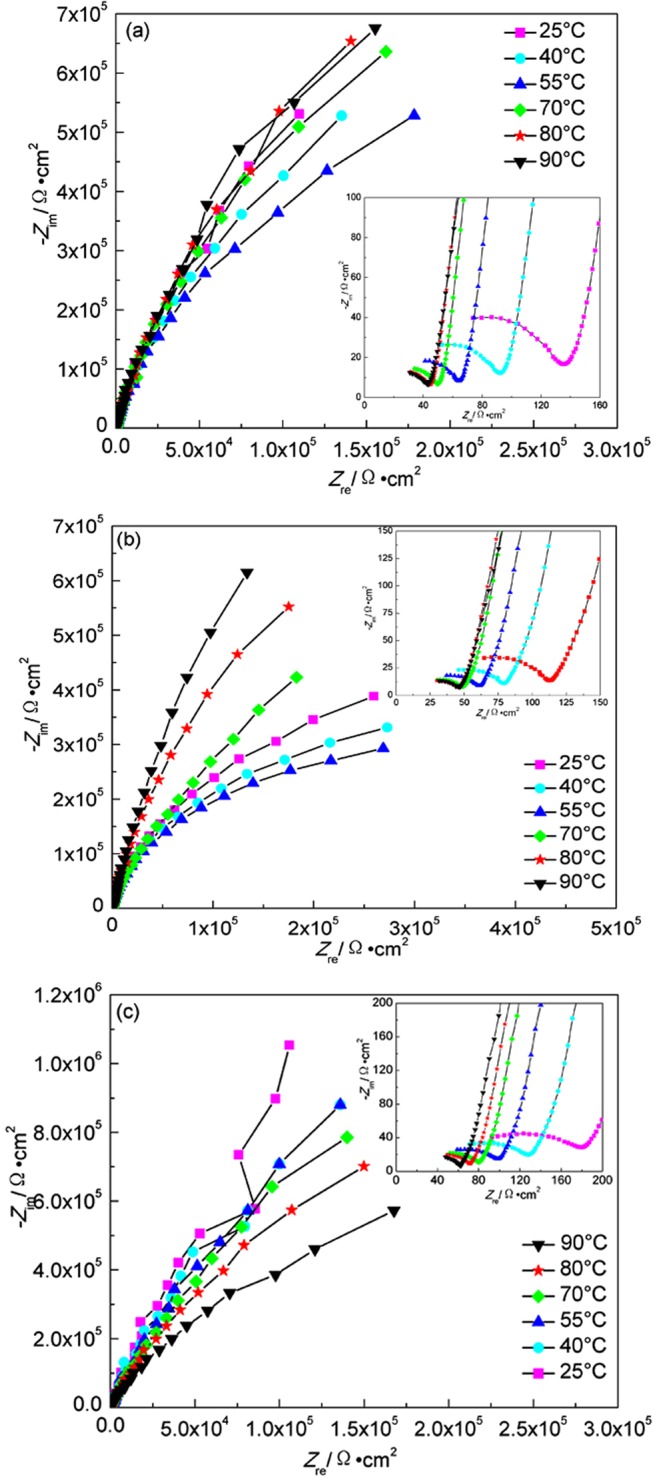
EIS plots of Ti grade 2 in highly compacted bentonite at different temperatures. (**a**) 20% water, temperature increase; (**b**) 25% water, temperature increase; (**c**) 30% water, temperature decrease.

Figure 9 shows the EIS plots of Ti grade 2 at different temperatures in highly compacted bentonite with 20% (a), 25% (b) and (c) 30% (saturation) water content. For Fig. 9(a,b), the radium of condensance arc both decrease at first up to minimum at 55 °C and then increase. At first, low oxygen content and low reaction rate at low temperatures lead to incomplete metal oxide film formation on the surface of Ti grade 2. The radius reduction of condensance arc with temperature rising results from the accelerating effect of temperature on the anodic and cathodic processes on the bare surfaces of the metal without oxide film coverage. Further increase of the temperature facilitates the oxygen diffusion and then the integrated oxide film formation on the surface, showing a radius of condensance arc increase again with increasing temperature.

For Fig. 9(c), the radium of capacitive arc shows a trend of increase with temperature decrease. The corrosion resistance of Ti grade 2 is much higher than that of A283-D steel. No visible corrosion product was observed after the measurement, indicating that the temperature is the main affecting factor controlling corrosion rate. The equivalent circuit shown in Fig. 8(a) was used to fit the EIS data of Ti grade 2 specimen. The fitted parameters are shown in Table 2.

**Table 2:** Fitted EIS results of Ti grade 2 in highly compacted bentonite with 30% water content at different temperatures.

Temperature(°C)	*R*_*s*_(Ω·cm^2^)	*R*_*b*_(Ω·cm^2^)	*R*_*ct*_(Ω·cm^2^)
90	35.45	30.28	6.30 × 10^6^
80	38.87	37.00	3.95 × 10^7^
70	41.34	43.49	1.46 × 10^9^
55	50.50	54.69	2.89 × 10^15^
40	62.98	73.69	2.76 × 10^15^
25	86.63	105.30	7.09 × 10^16^

It is observed that *R*_*s*,_ *R*_*b*_ and *R*_*ct*_ all increase with the decrease of temperatures in highly compacted bentonite saturated with water. The increase of *R*_*s*_ with the decrease of temperature results from the reduced mobility of the conductive ions, while the increase of *R*_*b*_ results from the gradual thickening of the surface film, although it is not visible. The much higher value of *R*_*ct*_ compared with that of A283-D steel indicates the much less corrosion rate of Ti grade 2. It can also be inferred from the increased *R*_*ct*_ with the decrease of temperature that the reduced corrosion rate is expected after a prolonged geological disposal of the container.

**Figure 10:**
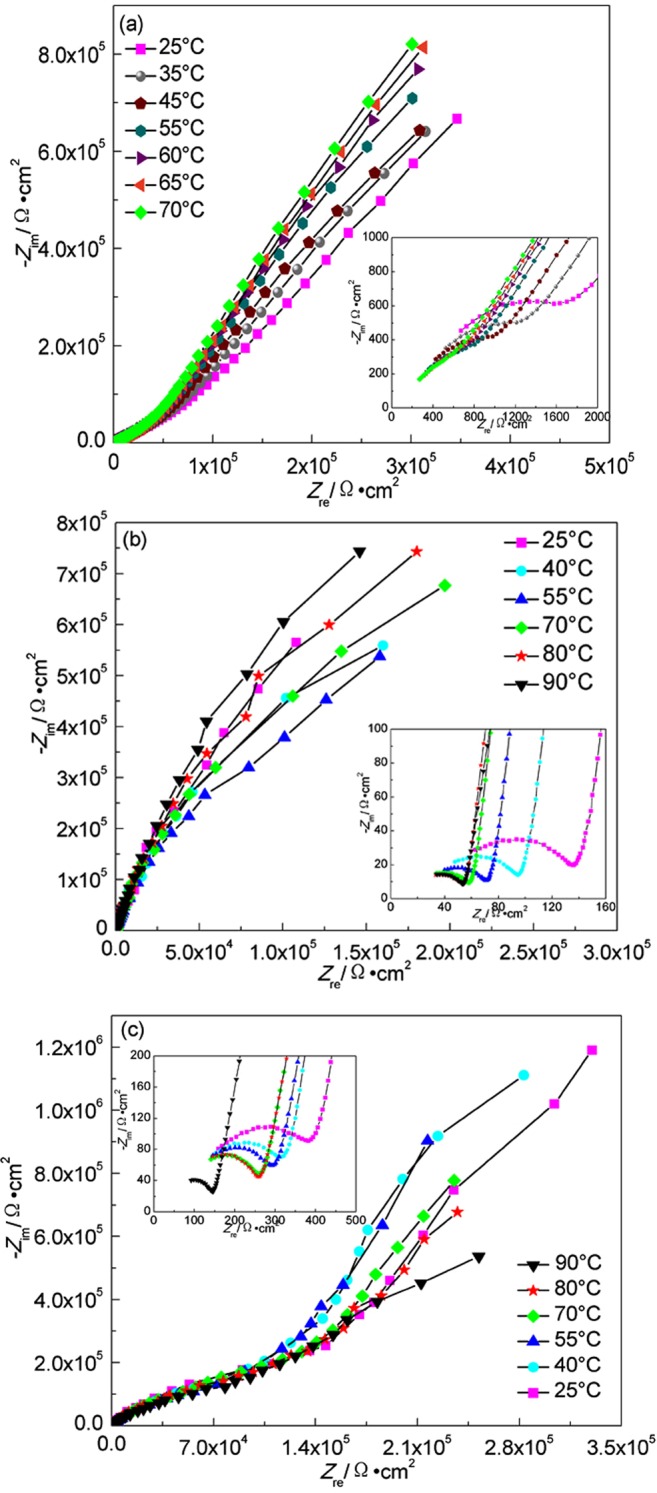
EIS plots of Ti grade 16 in highly compacted bentonite at different temperatures. (**a**) 20% water, temperature increase; (**b**) 25% water, temperature increase; (**c**) 30% water, temperature decrease.

The EIS plots of Ti grade 16 in highly compacted bentonite with water content below 30% are similar with that of Ti grade 2. While, there exist some differences for the EIS plots of Ti grade 16 in saturated bentonite (30% water content), which are shown in Fig. 10(c). There appear three capacitive reactance arcs corresponding to capacitance of the electric double layer between specimen and bentonite, capacitance of recrystallized film on corrosion product and the capacitance of corrosion product film. D. W. Shoesmith *et al*.^42^ believe that a recrystallized layer forms outside the initial passivation film due to internal stress induced by the difference between the molar volume of metal substrate and the passivation film, which explains the appearance of three capacitive reactance arcs. The equivalent circuit shown in Fig. 8(b) was used to fit the EIS data and the fitted parameters are shown in Table 3.

**Table 3:** Fitted EIS results of Ti grade 16 in highly compacted bentonite with 30% water content at different temperatures.

Temperature(°C)	*R*_*s*_(Ω·cm^2^)	*R*_*a*_(Ω·cm^2^)	*R*_*b*_(Ω·cm^2^)	*R*_*ct*_(Ω·cm^2^)
90	35.45	130.77	3.95 × 10^5^	3.96 × 10^6^
80	48.70	241.83	3.06 × 10^5^	1.45 × 10^7^
70	26.19	260.87	1.92 × 10^5^	1.67 × 10^7^
55	47.26	279.73	1.53 × 10^5^	1.70 × 10^7^
40	66.29	283.07	1.67 × 10^5^	1.85 × 10^7^
25	75.44	367.30	2.41 × 10^5^	1.13 × 10^7^

It can be seen from Table 3 that the resistance *R*_*b*_ of corrosion product layer formed at initial higher temperature is larger. But the induced internal stress in the corrosion product hinders its integrity because of the mismatch in molar volume between corrosion product and the metal substrate, resulting in the corrosion product layer resistance’s lowering in later temperature decreasing period. The trivial increase of *R*_*b*_ below 40 °C can be regarded as corrosion product accumulation. The increase of *R*_*a*_ results from the thickening of the recrystallized film. The charge transfer resistance *R*_*ct*_ shows a general increase with the decrease of temperature, indicating the decrease of corrosion rate as temperature decreases.

### Potentiodynamic polarization curves

**Figure 11:**
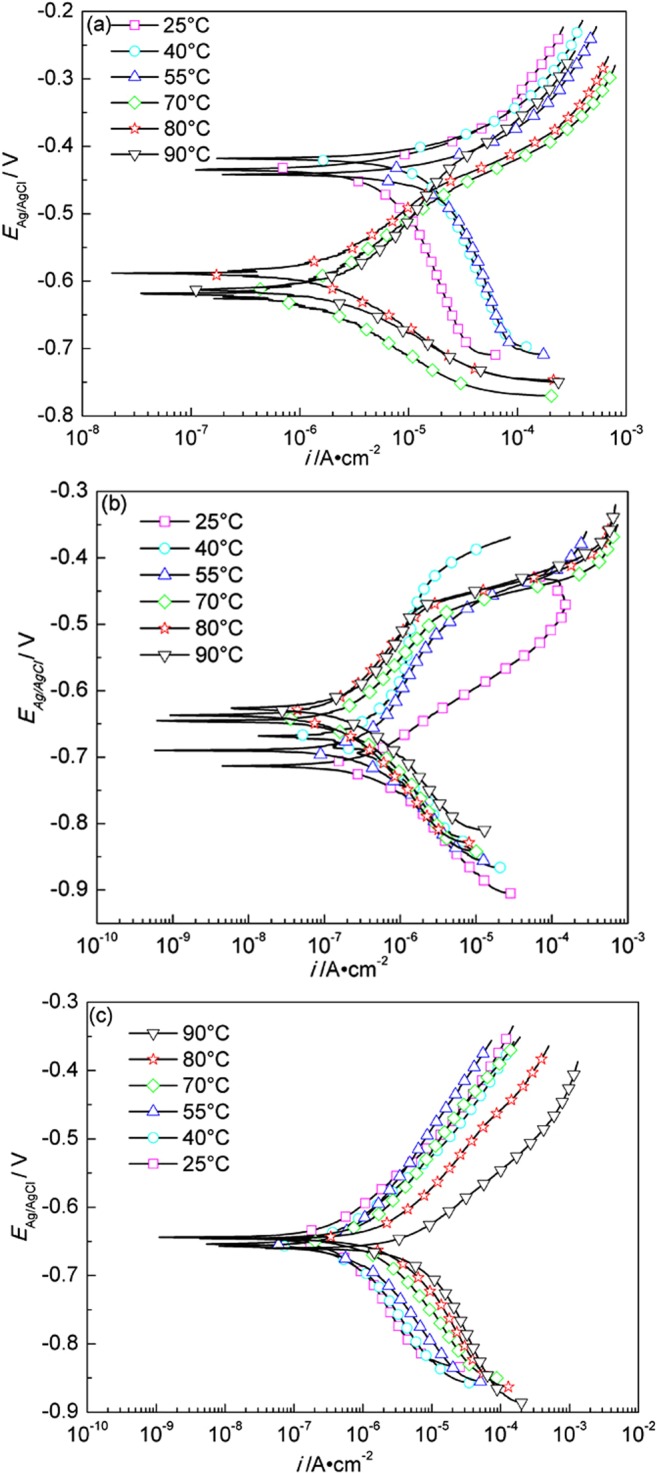
Tafel polarization curves of A283-D steel in highly compacted bentonite with 20% (**a**), 25% (**b**) and 30% (saturation) (**c**) water content at different temperatures.

Fig. 11 shows the Tafel polarization curves of A283-D steel at different temperatures in highly compacted bentonite with 20% (a), 25% (b) and (c) 30% (saturated) water content. For the cases of (a) and (b), a comparative complex regularity is shown. While, for the case of (c), the polarization curves shift monotonously towards left with temperature decrease, and it is more distinct at higher temperatures. The fitted results of polarization curves are shown in Table 4.

**Table 4:** Fitted results of polarization curves of A283-D steel in highly compacted bentonite at different temperatures (HCB-x% stands for highly compacted bentonite with x% water content).

System	*E*_*corr*_ vs. SCE/mV	*β*_*a*_/mV·dec^−1^	*β*_*c*_/mV·dec^−1^	*I*_*corr*_/μA·cm^−2^	Corrosion rate/mm·a^−1^
HCB-20%–25 °C	−478	209.04	−411.06	18.37	0.21
HCB-20%–40 °C	−463	185.71	−385.61	33.38	0.40
HCB-20%–55 °C	−395	235.40	−494.19	52.47	0.61
HCB-20%–70 °C	−666	86.89	−124.23	2.06	0.024
HCB-20%–80 °C	−637	78.33	−125.72	1.87	0.022
HCB-20%–90 °C	−663	229.83	−91.34	4.67	0.054
HCB-25%–25 °C	−756	72.92	−137.30	0.66	0.0077
HCB-25%–40 °C	−713	764.55	−101.23	1.09	0.013
HCB-25%–55 °C	−736	230.70	−145.25	0.70	0.0082
HCB-25%–70 °C	−693	133.93	−196.51	0.51	0.0060
HCB-25%–80 °C	−686	145.08	−159.86	0.30	0.0034
HCB-25%–90 °C	−676	204.95	−148.17	0.49	0.0057
HCB-30%–25 °C	−730	96.00	−173.03	0.77	0.0090
HCB-30%–40 °C	−744	92.51	−156.90	0.90	0.010
HCB-30%–55 °C	−749	151.14	−143.05	1.33	0.016
HCB-30%–70 °C	−742	145.34	−157.15	2.63	0.031
HCB-30%–80 °C	−751	121.51	−177.35	5.08	0.059
HCB-30%–90 °C	−760	82.81	−217.59	10.94	0.13

The corrosion potential in highly compacted saturated bentonite at every temperature is lower than that in the bentonite with lower water content in general, indicating that water is the prerequisite to form corrosive conditions in the bentonite. But the maximum corrosion rate doesn’t appear in the saturated bentonite (30% water content) but appears in the bentonite with 20% water content, showing the complexity of bentonite water content, temperature and O_2_-transport limited on metal corrosion^43,44^. Compared with previous work^45^, the corrosion rate of A283-D specimens in highly compacted bentonite is lower than that in simulated underground water. Backfill of bentonite is then beneficial in controlling the corrosion of nuclear waste containers.

**Figure 12:**
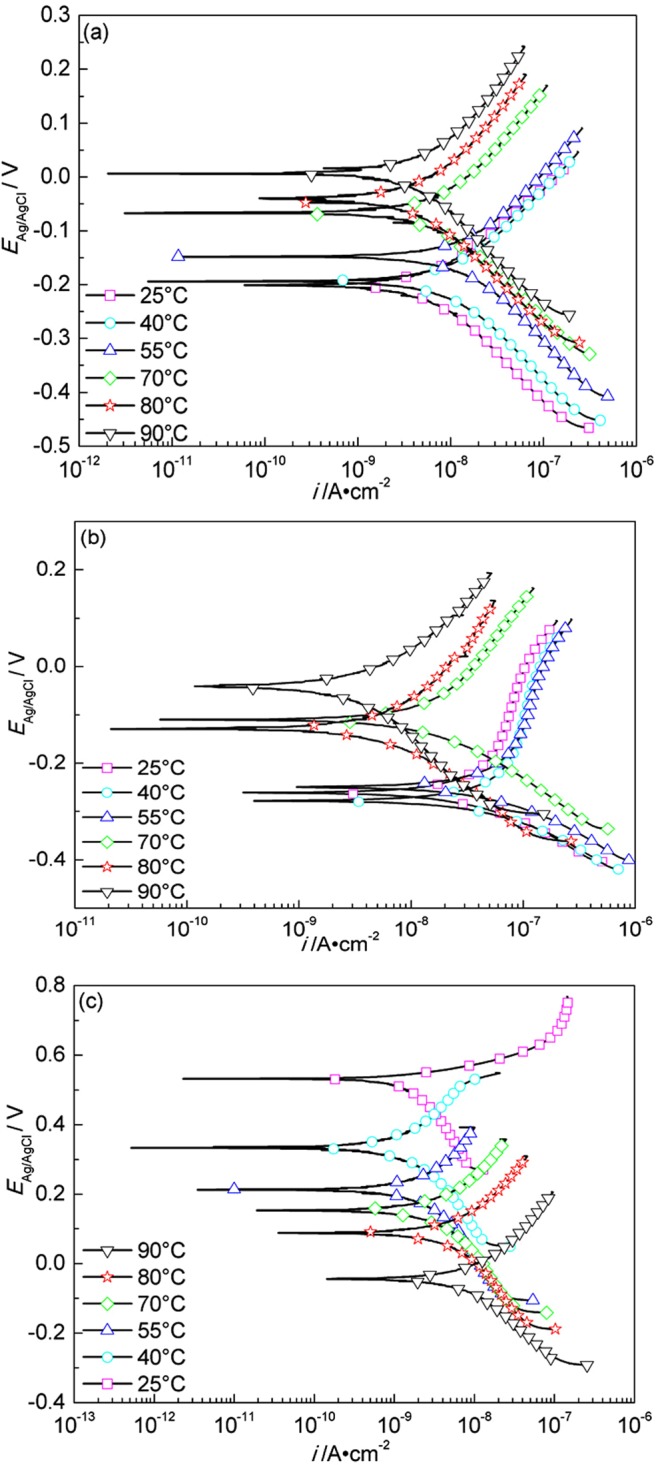
Tafel polarization curves of Ti grade 2 in highly compacted bentonite with 20% (**a**), 25% (**b**) and 30% (saturation) (**c**) water content at different temperatures.

Figure 12 shows the Tafel polarization curves of Ti grade 2 at different temperatures in highly compacted bentonite with different water content. The fitted parameters of polarization curves are shown in Table 5.

**Table 5:** Fitting results of polarization curves of Ti grade 2 in highly compacted bentonite at different temperatures (HCB-x% stands for highly compacted bentonite with x% water content).

System	*E*_*corr*_ vs.SCE/mV	*β*_*a*_/mV·dec^−1^	*β*_*c*_/mV·dec^−1^	*I*_*corr*_/10^−3^μA·cm^−2^	Corrosion rate/10^−5^mm·a^−1^
HCB-20%–25 °C	−201	153.82	−180.67	8.05	7.00
HCB-20%–40 °C	−194	174.34	−183.22	12.80	11.13
HCB-20%–55 °C	−148	192.46	−191.08	19.02	16.54
HCB-20%–70 °C	−73	207.76	−172.98	10.65	9.26
HCB-20%–80 °C	−44	222.18	−172.04	6.45	5.61
HCB-20%–90 °C	7	240.74	−189.73	7.09	6.17
HCB-25%–25 °C	−261	758.89	−136.78	56.18	48.85
HCB-25%–40 °C	−278	929.61	−136.63	82.14	71.42
HCB-25%–55 °C	−249	812.09	−132.33	86.70	75.39
HCB-25%–70 °C	−117	321.25	−159.60	22.39	19.47
HCB-25%–80 °C	−121	303.14	−170.87	10.94	9.51
HCB-25%–90 °C	−59	248.32	−226.12	6.44	5.60
HCB-30%–25 °C	515	111.40	−306.77	3.07	2.67
HCB-30%–40 °C	332	196.90	−342.76	1.82	1.58
HCB-30%–55 °C	217	335.92	−352.29	3.70	3.21
HCB-30%–70 °C	154	302.52	−357.97	6.00	5.22
HCB-30%–80 °C	88	280.97	−318.48	8.36	7.27
HCB-30%–90 °C	−34	273.12	−199.69	13.72	11.93

It can be seen that the corrosion potentials at different temperatures are in general more positive in highly compacted saturated bentonite than those in bentonite with lower contents of water, which is in agreement with the section of OCP measurement. Higher water content facilitates the formation of a uniform electrolyte film at the surface between Ti grade 2 specimen and bentonite and hence the formation of a uniformed corrosion product layer, retarding further corrosion reactions. Similar to A283-D steel, the corrosion rate of Ti grade 2 specimens in highly compacted bentonite is lower than that in simulated underground water^45^. The maximum corrosion rate of Ti grade 2 appears not in saturated bentonite but in the bentonite with 25% water content. Water and oxygen are the critical factors affecting corrosion. Saturated bentonite reduces the trapped oxygen by reducing the voids in it, while lower water content in the bentonite impedes the progress of electrochemical processes. Water content around 25% happens to be the critical water content with certain amount of trapped oxygen available and the water content is just enough to form appropriate coverage of electrolyte on the surface of specimen to facilitate the electrochemical process, and consequently a higher corrosion rate shows up.

**Figure 13:**
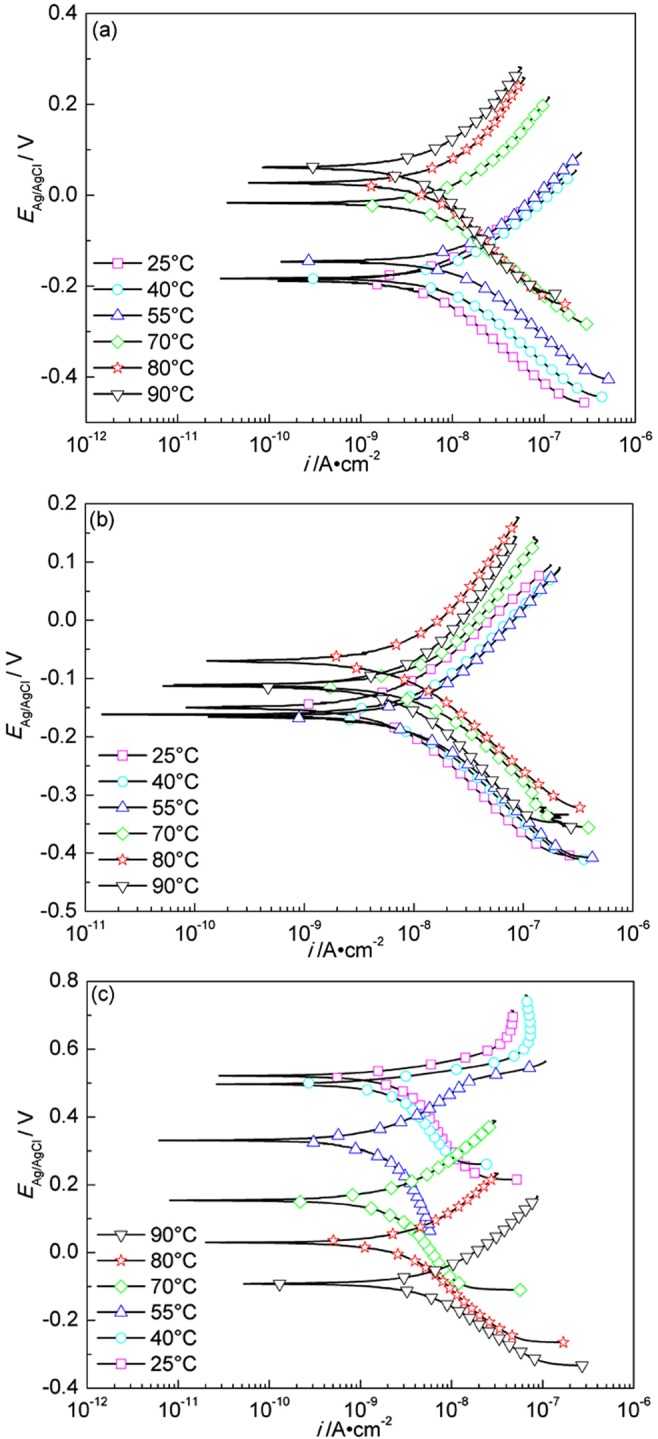
Tafel polarization curves of Ti grade 16 in highly compacted bentonite with 20% (**a**), 25% (**b**) and 30% (saturation) (**c**) water content at different temperatures.

Figure 13 shows the Tafel polarization curves of Ti grade 16 at different temperatures in highly compacted bentonite with different water content. The fitted parameters of polarization curves are shown in Table 6. Similar to Ti grade 2, the corrosion potentials at different temperatures are in general more positive in highly compacted saturated bentonite than those in bentonite with lower contents of water, and the corrosion rate of Ti grade 16 specimens in highly compacted bentonite is also lower than that in simulated underground water^45^. The maximum corrosion rate of Ti grade 16 appears in the bentonite with 25% water content as well. The explanations for Ti grade 2 can also explain the results of Ti grade 16. For Ti grades 2, 16, the reaction mechanism is still unclear and need to be further studied.

**Table 6:** Fitting results of polarization curves of Ti grade 16 in highly compacted bentonite at different temperatures (HCB-x% stands for highly compacted bentonite with x% water content).

System	*E*_*corr*_ vs.SCE/mV	*β*_*a*_/mV·dec^−1^	*β*_*c*_/mV·dec^−1^	*I*_*corr*_/10^−3^μA·cm^−2^	Corrosion rate/10^−5^mm·a^−1^
HCB-20%–25 °C	−189	155.18	−179.17	6.93	5.96
HCB-20%–40 °C	−183	172.95	−176.19	11.75	10.11
HCB-20%–55 °C	−146	188.20	−182.64	16.70	14.36
HCB-20%–70 °C	−29	240.73	−166.92	12.14	10.44
HCB-20%–80 °C	27	283.82	−230.30	10.58	9.10
HCB-20%–90 °C	63	249.74	−249.92	7.57	6.51
HCB-25%–25 °C	−150	170.23	−185.00	8.06	6.93
HCB-25%–40 °C	−162	191.29	−181.84	12.69	10.91
HCB-25%–55 °C	−166	210.30	−190.75	16.13	13.87
HCB-25%–70 °C	−114	254.03	−173.81	16.83	14.48
HCB-25%–80 °C	−74	268.11	−174.47	13.07	11.24
HCB-25%–90 °C	−145	293.06	−190.19	11.36	9.77
HCB-30%–25 °C	508	145.18	−345.69	4.28	3.69
HCB-30%–40 °C	507	317.12	−637.43	7.72	6.64
HCB-30%–55 °C	319	193.97	−600.59	2.64	2.27
HCB-30%–70 °C °C	145	212.71	−383.44	3.20	2.75
HCB-30%–80 °C	30	230.38	−263.88	4.36	3.74
HCB-30%–90 °C	−90	241.14	−187.82	8.21	7.06

### Corrosion rate estimation over geological time scale after disposal

**Figure 14:**
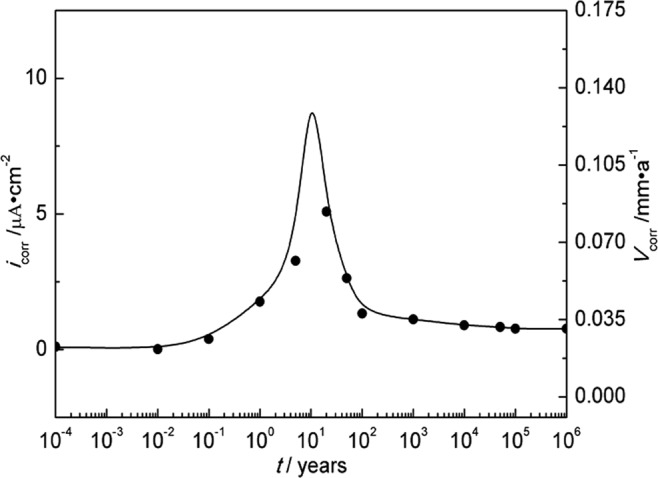
Corrosion rate of A283-D steel in deep geological disposal environment over an expected disposal period up to 10^6^ years in Beishan area of China.

**Figure 15:**
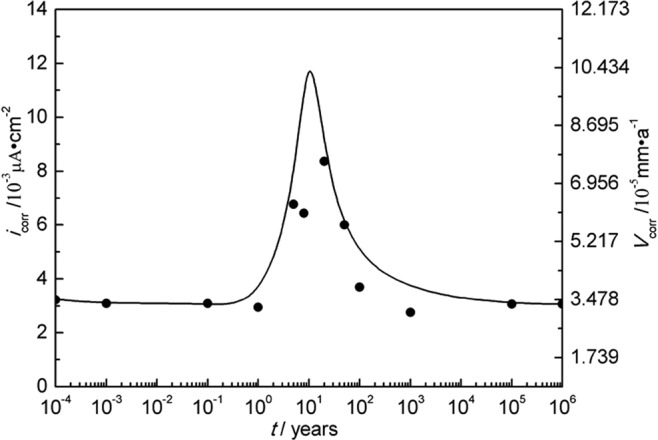
Corrosion rate of Ti grade 2 in deep geological disposal environment over an expected disposal period up to 10^6^ years in Beishan area of China.

**Figure 16:**
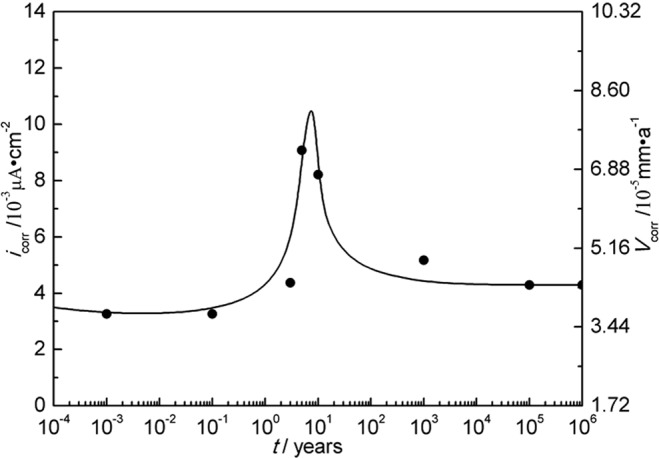
Corrosion rate of Ti grade 16 in deep geological disposal environment over an expected disposal period up to 10^6^ years in Beishan area of China.

The temperature and water content near the interface of nuclear waste containers/bentonite evolve with time after disposal. The geological time scale of corrosion rate estimation was done based on the model in Fig. 3. According to Fig. 3, temperature and water content correspond to different geological disposal times. At the year of 10^−4^, the temperature is 25 °C and the water content is 25%. The corrosion rate at the temperature of 25 °C and the water content of 25% was acquired by fitting polarization curves. Therefore, the corrosion rate corresponds to the different geological disposal times. The rest data can be deduced in this way. The water content is up to saturation especially ten years later and no longer changes, with temperature changing only. According to the predicted geological evolution of temperature and water content^30^ and the measured corrosion rates of A283-D steel and Ti grades 2, 16 in bentonite with different water content at different temperatures shown in Tables 4–6, the scenarios of corrosion rates over disposal periods were estimated and the results are shown in Figs 14–16 respectively. Of all the three materials studied, there exhibit corrosion rate peaks between 1 and 100 years. The corrosion rates increase firstly, reach the maximum and then decrease gradually to a relatively stable value. Integrate Figs 14–16 and Table 7 was acquired. Table 7 shows the estimated corrosion depth of the 3 materials over different disposal periods. Because of the higher corrosion rate of carbon steels, carbon steel is valid for the fabrication of waste containers for low and medium level radioactive waste disposal. While, titanium and its alloy are suitable for HLRW disposal due to their excellent corrosion resistance over large time scale. Therefore, only from the perspective of corrosion rate, titanium and its alloys have much higher reliability than A283-D steel as container materials in the long term deep geological disposal environment. However, for deep geological disposal of HLRW container, there are indeed many factors affecting it. In the research, we focus on the general corrosion behavior of container as a function of water contents and temperature. Therefore, some assumptions must be given. Firstly, it is assumed that the corrosion type of container is general corrosion and it is indeed general corrosion from laboratory measurements. Secondly, it is assumed the container is thick enough and still existing up to 10^6^ years without complete corrosion penetration. Thirdly, hydrogen embrittlement and radiation effect of HLRW can be neglected. The work conducted by R. J. Winsley *et al*. confirmed that the HLRW radiation intensity on corrosion has negligible effect^46^. The corrosion of containers will experience a long process, during which the surrounding environment changes from aerobic to unaerobic, hydrogen evolution reaction becomes the main cathodic reaction. These two points are our next stage of research. Lastly, it is assumed that geological movement, bacteria and other factors are not affected. Meanwhile, it should be noted that the current research is based on short term electrochemical techniques. The complex corrosion behavior of materials over large time scale can be expected. The effects of corrosion products scale formation, radiation and bacteria etc. in the repository may all play a role in the corrosion process. Much work needs to be done to get a clearer scenario of corrosion development over geological time scale.

**Table 7:** Estimated corrosion depth of A283-D steel and Ti grades 2, 16 over different disposal periods.

Time/years	10	100	1000	10000	50000	1000000
A283-D steel-corrosion depth/mm	0.54	4.0	16.8	122.3	525.5	9058.2
Ti-2-corrosion depth/mm	0.00065	0.0034	0.034	0.53	2.9	38.7
Ti-16-corrosion depth/mm	0.00064	0.0032	0.032	0.51	2.9	38.6

## Methods

### Materials and corrosive environment

Carbon steel A283-D, Ti grade 2 and Ti grade 16 (ASTM) are used as the experimental materials, whose chemical compositions are shown in Tables 8–10 respectively.

**Table 8:** The chemical composition of A283-D (wt%) (ASTM).

C	Si	Mn	P	S	Fe
0.14	0.1	0.41	0.015	0.038	balance

**Table 9:** The chemical composition of Ti grade 2 (wt%) (ASTM).

Fe	C	N	H	O	Ti
0.3	0.08	0.03	0.015	0.25	balance

**Table 10:** The chemical composition of Ti grade 16 (wt%) (ASTM).

Fe	C	N	H	O	Pd	Ti
0.3	0.08	0.03	0.015	0.18	0.06	balance

Columnar specimens with dimensions of ϕ10 × 10 mm were obtained by wire-electrode cutting across the experimental material bars. After being cleaned with absolute ethanol and dried in a flow of cool air, the specimens were packed in heat shrink tube with heating to make sure the tubing encapsulated the side surface of them tightly. Insulated copper conductor was connected on one end of the specimen and sealed with epoxy resin. Another end is the working surface with area of 0.785 cm^2^. Prior to the tests, specimens were grounded sequentially with emery paper from 240# to 1000#, cleaned with absolute ethanol, and dried in a flow of cool air.

**Figure 17:**
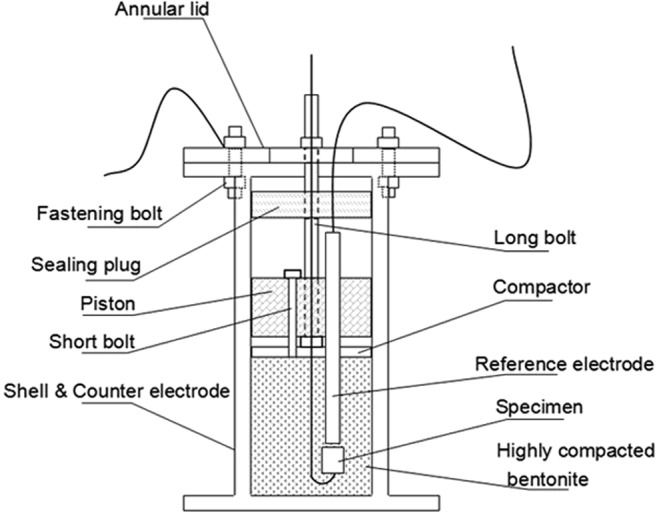
Schematic diagram of the apparatus for highly compacted bentonite preparation and the electrochemical measurement in such environment.

The simulated underground water was prepared according to the typical underground water compositions of Beishan area^47^ as shown in Table 11, and pH was adjusted to 7.5 before experiments. The composition of Na-bentonite with pH being 7.8 from Gaomiaozi area of inner Mongolia is shown in Table 12. The bentonite was dried at 105 °C to a constant weight before use. In order to simulate a real geological disposal environment, the bentonite was not deaerated at the start of the experiment. Therefore, there is a portion of oxygen sealed in bentonite. The bentonite with different water content including 15%, 20%, 25% and 30% (saturation) corresponding to different deep disposal years was prepared with simulated underground water by spraying and curing method. The highly compacted bentonite whose density is 1.7 g/cm^3^ is prepared by layer after layer pressing with the apparatus shown in Fig. 17. The density is the wet density. The apparatus is also the electrochemical testing unit with three-electrode system built in.

**Table 11:** The typical chemical compositions of underground water in Beishan area of China (mg/L).

Na^+^	K^+^	Ca^2+^	Mg^2+^	HCO_3_^−^	Cl^−^	SO_4_^2−^	NO_3_^−^
1170.07	20	57.37	219.43	96.37	1261.33	1259.67	27.10

**Table 12:** Chemical compositions of Gaomiaozi-Na-bentonite (mass fraction/%).

Component	Al_2_O_3_	SiO_2_	P_2_O_5_	CaO	K_2_O	TiO_2_	FeO	TFe_2_O_3_	MgO	Na_2_O	MnO	loss on ignition
Mass fraction	14.24	68.40	0.05	0.99	0.68	0.14	0.26	2.53	3.31	1.62	0.036	7.67

The near field measurements and simulating analysis showed that the environment around the nuclear waste containers was unaffected by the underground water infiltration just after the backfill of bentonite but mainly affected by the heat release from nuclear waste decay^28,30–33,47,48^. A significant increase in temperature leads to a decrease in water content in bentonite, but it will then be complemented by the infiltration of groundwater and will be saturated finally. The water content increases obviously in about 3 years and then rapidly afterwards in general. The saturation will be reached in about 10 years, and the water content will be almost constant in the following years. Considering the differences in simulation processes and the time scale of study, the simulation results of Japan is considered reasonable as the bentonite water content and temperature evolution near the surface of nuclear waste containers^48^.

The temperatures were controlled from 25 to 90 °C with thermostat water bath The temperature control before the saturation of bentonite by groundwater was done by gradient increase method. While, when the bentonite was saturated (30% water content), gradient cooling was adopted to simulate the temperature decrease at different geological ages.

### Electrochemical measurements

A three-electrode system was employed in the electrochemical measurements with A283-D and Ti grades 2, 16 columnar specimens being working electrodes respectively, stainless steel cylinder filled with compacted bentonite being counter electrode and solid Ag/AgCl electrode being the reference as illustrated in Fig. 17.

OCP, EIS and PC were all conducted through a CHI604B electrochemical analyzer. The EIS measurements were performed over the frequency range from 10^5^ to 10^−2^ Hz at the corrosion potential with the ac perturbation amplitude of ±10 mV. ZSimpWin software was used to analyze the impedance spectra to get the parameters of each element of equivalent circuit. The PC measurements were carried out by applying over-potentials from −250mV to 250 mV with respect to the corrosion potential at a scan rate of 0.167 mV/s.

## Conclusions

The corrosion behavior of A283-D steel and Ti grades 2, 16 that serve as competitive candidate materials of nuclear waste containers for deep geological disposal in highly compacted bentonite with different simulated underground water content was studied. Meanwhile, the corrosion rate evolution of these materials over long deep geological timescales was estimated.

1. The corrosion rates of the 3 materials in highly compacted bentonite are lower than those obtained earlier in simulated underground water. The backfill of bentonite is beneficial in controlling the corrosion of nuclear waste containers.
2. The corrosion rates of Ti grades 2, 16 are far lower than that of A283-D steel in highly compacted bentonite under deep disposal conditions over geological time scales. Therefore titanium and its alloys have much higher reliability than A283-D steel as container materials in the long term deep geological disposal environment based on corrosion rate considerations.

Moreover, this research can also provide some references for other researchers who deal with similar conditions, such as steel in concrete of bridge, underground pipelines, and even the dental and orthopedic implants etc.

## Data Availability

The datasets generated during and/or analysed during the current study are available from the corresponding author on reasonable request.
